# Synthesizing Electronic Health Records for Predictive Models in Low-Middle-Income Countries (LMICs)

**DOI:** 10.3390/biomedicines11061749

**Published:** 2023-06-18

**Authors:** Ghadeer O. Ghosheh, C. Louise Thwaites, Tingting Zhu

**Affiliations:** 1 Department of Engineering Sciences, University of Oxford, Oxford OX1 3PJ, UK; 2 Oxford University Clinical Research Unit (OUCRU), Ho Chi Minh City 710400, Vietnam; 3 Centre for Global Health and Tropical Medicine, University of Oxford, Oxford OX3 7LG, UK

**Keywords:** electronic health records, synthetic data, low-middle-income countries (LMICs), hospital-acquired infections

## Abstract

The spread of machine learning models, coupled with by the growing adoption of electronic health records (EHRs), has opened the door for developing clinical decision support systems. However, despite the great promise of machine learning for healthcare in low-middle-income countries (LMICs), many data-specific limitations, such as the small size and irregular sampling, hinder the progress in such applications. Recently, deep generative models have been proposed to generate realistic-looking synthetic data, including EHRs, by learning the underlying data distribution without compromising patient privacy. In this study, we first use a deep generative model to generate synthetic data based on a small dataset (364 patients) from a LMIC setting. Next, we use synthetic data to build models that predict the onset of hospital-acquired infections based on minimal information collected at patient ICU admission. The performance of the diagnostic model trained on the synthetic data outperformed models trained on the original and oversampled data using techniques such as SMOTE. We also experiment with varying the size of the synthetic data and observe the impact on the performance and interpretability of the models. Our results show the promise of using deep generative models in enabling healthcare data owners to develop and validate models that serve their needs and applications, despite limitations in dataset size.

## 1. Introduction

Clinical decision support systems (CDSS) are important tools to promote optimal patient care, safety, and use of resources. In low and middle-income countries (LMICs), where an estimated 8,000,000 deaths occur every year as a result of low-quality (but accessible) care, such systems have the potential to make a huge impact [1]. Developing CDSS applications using electronic health records (EHRs) and machine learning (ML) techniques has gained increased interest from the research community [2]. Despite the promising results of many of these applications, the performance of ML models is highly dependent on the availability of training data [3,4]. ML models tend to be data hungry, and can easily overfit and under-perform when trained on a small dataset [3,4].

Most CDSS have been developed in high-income countries making use of huge datasets available from EHRs [5,6]. Consequently, they fail to support decision-making in many diseases that are prevalent in low-resource settings, resulting in an unmet need for ML research applications that are developed and validated for low-resource settings. Even if CDSS address issues common to all resource settings, those developed from high-income datasets are usually unsuitable for direct deployment in LMIC settings due to differences in the prevalence of diseases and demographic distribution [7,8,9], and require adaptation using data from these populations [10]. For example, hospital-acquired infections (HAI) are well-established markers of healthcare quality as well as being significant causes of mortality, and morbidity in patients throughout the world. They are a particular concern in LMICs and CDSS predicting those at risk of HAI would be of huge value in improving patient outcomes. However, as HAI is closely linked to the local context, the development of these is particularly reliant on high-quality local data.

EHRs are rarely available in LMICs [11] and their healthcare systems often suffer from infrastructural and diagnostic capacity constraints [12], frequent changes in strategic healthcare policies, and political instability [13], all of which could impact the quantity and quality of routine healthcare data collected from such clinical settings. Manual collection of high-quality large-scale data is unfeasible in terms of cost and personnel. The dependence on data slows down the optimal development and utilization of CDSS, specifically in resource-constrained clinical settings.

Many of the current medical statistics and data-driven models rely on methods such as Synthetic Minority Oversampling TEchnique (SMOTE) which oversample the training data, especially in imbalanced settings. Oversampling methods could introduce flawed correlations and dependencies between samples and result in limited data variability [14], all of which could severely underperform in testing environments. Recent works in deep learning research have proposed generative models that learn the underlying data distribution and generate realistic-looking data while preserving the privacy of the original samples. These deep generative models, including Generative Adversarial Networks (GANs) and Variational AutoEncoders (VAEs) [15,16] have been originally proposed and validated for the imaging domain where quantitative and qualitative evaluation by experts could not differentiate the real images from those generated by the models. Despite being very relevant and highly needed, using deep generative models for synthesizing EHRs for low-resource clinical applications is often not discussed nor motivated in most proposed works [17].

To this end, this paper proposes synthetic data as a solution for developing models based on small datasets collected from LMIC countries. To do so, we train a GAN-based model to learn the underlying data distribution and generate synthetic samples that could be utilized for training purposes. Specifically, we utilize a small already published dataset (364 patients) collected from an Intensive Care Unit in Vietnam [18], with variables collected at admission and a binary outcome indicating if the patient received a hospital-acquired infection. With the increased burden of antimicrobial resistance, especially in LMICs, it is vital to develop risk scores to predict the probability of developing such infections. This could allow the clinical staff to take anti-septic measures, reduce unnecessary antibiotics prescriptions, and introduce timely interventions to prevent prolonged lengths of stays. The proposed method provides a plausible solution that could be used for developing diagnostic models despite data scarcity in LMICs. Our contributions could be summarized as follows.

1. Deep generative models for LMICS. For the first time, we demonstrate the feasibility of using generative models for synthesizing data that is used to develop ML models from small datasets from LMIC healthcare settings.
2. Comprehensive data utility evaluation. We evaluate the utility of the synthetic data in comparison to other commonly used approaches and demonstrate a superior performance using models trained on synthetic data. We also showcase the impact of synthetic tabular data size on the performance of the predictive model in a series of experiments where the synthetic data training size is varied.
3. Interpretability analysis: We conduct a post-hoc SHapley Additive exPlanations (SHAP) interpretability analysis to investigate the impact of using various training sets on the feature importance in the test set predictions, which is a new approach for evaluating deep generative models for EHRs.

The structure of the paper is as follows. In the methods, we first describe the dataset used in the study followed by an explanation of the model used to generate the synthetic data. The other subsections in the Section 2 discuss the predictive modelling task and the baseline methods used for comparing the performance of the proposed model followed by an overview of the interpretability analysis. In the Section 3 we present the performance of the models and the feature importance analysis findings. In the Section 4, we interpret the findings, discuss the limitations and strengths of the work and outline the future outlook for related research directions.

## 2. Materials and Methods

### 2.1. Dataset Description

The data used in this work is collected from Ho Chi Minh City Hospital for Tropical Diseases, Vietnam, and released for open access [18]. The patients included in this study totalled 364 individuals who were all admitted to the ICU and stayed at least 2 days. The included variables are those readily available at the admission of ICU, which we categorize into co-morbidities, demographics, and admitting diagnosis. The admission diagnosis included one of five categories: (1) Tetanus, (2) Sepsis, (3) Local Infections, (4) Dengue, and (5) Internal Medicine disease. According to the original study documentation, the local infections included cases of pneumonia, cellulitis, urinary tract infection, and spontaneous bacterial peritonitis while the internal medicine diseases included kidney failure, myocarditis, myocardial infarction, malignant hypertension, diabetic ketoacidosis, and epilepsy [18]. The outcome of interest is a binary label indicating if the patient acquired an infection during their ICU stay. The included acquired infections in the dataset were pneumonia, bloodstream infection, and urinary tract infection, all of which were defined according to the Centers for Disease Control and Prevention Criteria 2014 [19].

### 2.2. Synthetic Data Generation

To evaluate the feasibility of using synthetic data as a training set, we apply a random stratified train-test split for our data to obtain separate training and test sets. We use a 70–30 split, which is a common choice for various machine learning studies [20], where the training data is used to train the generative model and the held-out test set is used to evaluate the performance of the downstream predictive model. While there are various generative models such as VAEs, we focus on GANs as they generate higher fidelity data and demonstrate higher performance in downstream predictive tasks [21]. Furthermore, VAEs are better suited for imaging [22] or time-series tasks [23] and less commonly used for generating tabular particularly discrete data such as our dataset. For this purpose, the training set is used to train the GAN model for tabular data, namely medGAN [24]. medGAN is considered one of the early works that adapted GANs for tabular EHR data, where the authors proposed an autoencoder to address the original GAN architecture’s incompatibility with dealing binary and discrete features. Upon training the GAN model the size of synthetic data is determined at inference time.

### 2.3. Predictive Modelling Task and Baselines

The generated synthetic data is used to train a simple machine learning model to predict hospital-acquired infections during the ICU stay of the patient. Three different types of machine learning models were evaluated, which were Random Forest [25], Support Vector Machines (SVM) [26], and K-Nearest Neighbour (KNN) [27], respectively. The choice of the three models is motivated by their relative simplicity, with often comparable performance to many advanced models, making them a good candidate for deployment in hospitals in LMICs. We compare the performance of the models trained on the synthetic data to those trained on the (1) original small training set and (2) oversampled training data using SMOTE. To better understand the impact of the synthetic data size on the predictive model performance, we train the GAN model to synthesize data of various sizes at inference. The synthesized data is then used to train the predictive model, where the performance is compared to that of models trained with original and oversampled data. Each of the machine learning models was trained using 3-Fold Stratified K-Fold validation, to choose the best hyperparameters using GridSearch to make the predictions on the held-out test set. The used hyperparameter ranges are included in the Supplementary Material Table S1. The final performance is reported on the held-out test set in terms of Area Under the Receiver Operating Characteristic Curve (AUROC) [28], Area Under the Precision-Recall Curve (AUPRC) [29], and balanced accuracy with confidence intervals computed using bootstrapping with 1000 iterations. While there are a variety of metrics that can be reported for predictive models (e.g., precision, recall, specificity) [30,31], our choice of AUROC and AUPRC was driven by their ability to summarise the trade-off between commonly reported metrics at various thresholds. For instance, the AUROC metric quantifies the trade-off between specificity and sensitivity at various thresholds [32], while AUPRC summarizes the trade-off between precision and recall at various thresholds [29]. We also choose to report balanced accuracy along with AUROC and AUPRC as they are more robust and indicative of the performance in the presence of imbalanced labels such as our dataset and outcome of interest when compared to normal metrics such as accuracy. Reporting metrics such as AUROC and AUPRC is a common practice in machine learning models [33,34], which can make it easier to interpret the findings and reduce the over-optimistic results of a single metric on its own.

**Figure 1:**
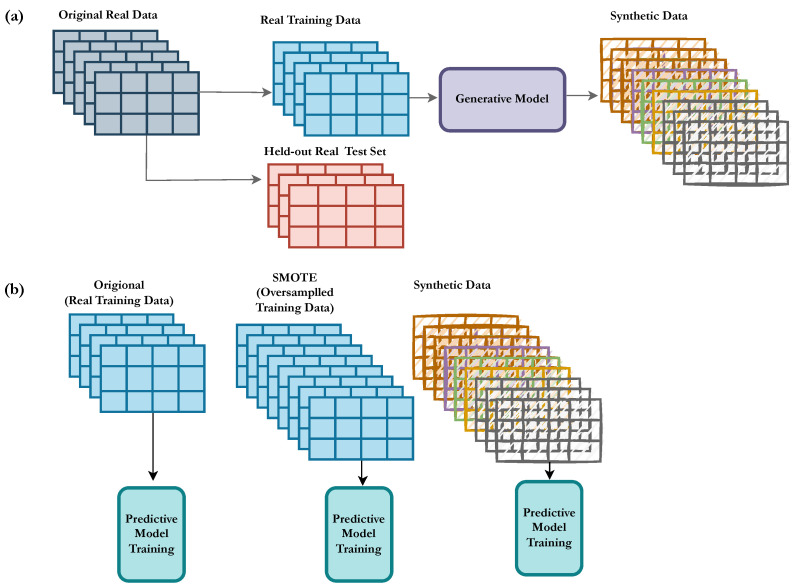
Overview of the proposed model trained on the synthetic data. (**a**) The dataset is split into training and a held-out test set. The training set is used to train the deep generative model that generates synthetic data. (**b**) A predictive model is trained in three different setups, (1) original, (2) SMOTE, and (3) synthetic data, which are evaluated on the held-out test set and compared in terms of the performance metrics.

The predictive modelling and data preparation was performed using Python (version 3.7) and the predictive models were trained using the scikit-learn package implementation. An overview of the predictive modelling and evaluation of our approach is presented in Figure 1.

### 2.4. Interpretability Analysis

In addition to reporting the performance of the models, we also evaluate the impact of using the various training sets on the model by conducting feature importance and interpretability analysis using post-hoc SHapley Additive exPlanations (SHAP) [35]. We use SHAP as the method to conduct the interpretability analysis due to its relative simplicity in interpreting the values, computational efficiency, and compatibility with a wide range of models. SHAP values are derived from a game theoretic basis, where the goal is to explain the ML model’s predictions for each instance by calculating the contribution of each of the features to the prediction. While SHAP values are computed for each of the instances separately, they are often reported in their aggregated format for all the features across all the samples. In this analysis, we report the mean absolute SHAP value for all the features, which indicates the relative importance of the feature in terms of the impact on the prediction across the test set. Specifically, we run the SHAP analysis for the models trained using (1) original (2) SMOTE, (3) synthetic data, using random forest classifier, and compare the relative importance of features. We conduct the analysis using SHAP open-source package, particularly the SHAP tree explainer [36], which works for tree ensemble models such as random forest used for the predictive analysis in this work.

## 3. Results

### 3.1. Predictive Modelling Task

The original data used to develop our predictive modelling is composed of 364 unique patients with a positive outcome prevalence of 23.6%. The population was 66.48% females with 39.01% of patients between 45 and 60 years old. We describe the statistical distribution of our dataset in terms of outcomes and included features in Table 1. In Table 2, we present the results of the models trained on the original training data (70% of the original data), the oversampled data, and synthetic data of various sizes, respectively.

**Figure 2:**
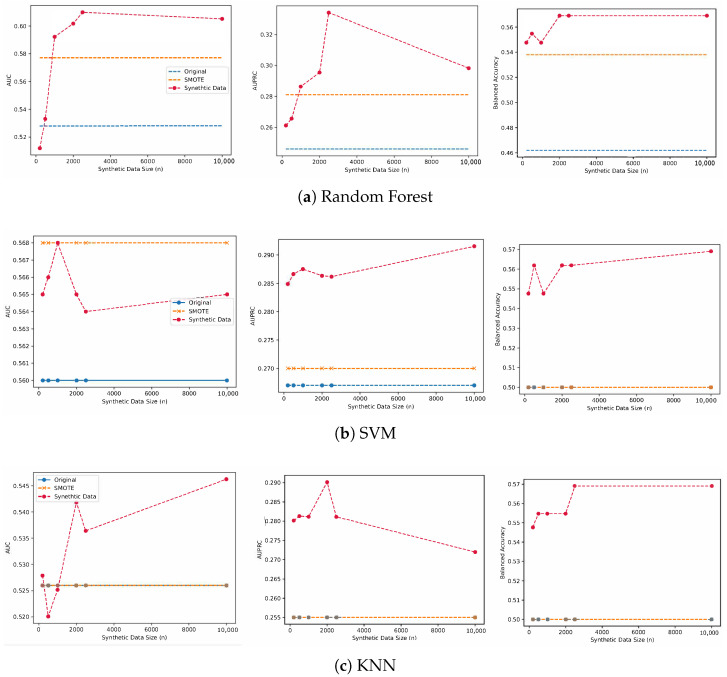
The performance of the predictive model trained using synthetic data of various sizes, SMOTE, and the original training set.

In general, the models trained on synthetic samples of a size greater than 500 consistently outperformed the model trained using the original data as well as the model trained on the oversampled data by SMOTE across the three classifier types. For the random forest model, the original model achieved a performance of 0.528 in AUROC, compared to 0.577 for the SMOTE baseline. On the other hand, the models trained on synthetic data outperformed the other baselines, with a performance of 0.610, 0.344, and 0.596 for AUROC, AUPRC and balanced accuracy, respectively. The models trained on the original data and SMOTE were first outperformed by the model trained with 1000 synthetic samples in terms of AUROC and AUPRC, where it also achieved the highest balanced accuracy of 0.592. We notice that performance gains after increasing the synthetic data size from 1000 to 10,000 are minimal, where the balanced accuracy did not change, with minor changes observed in AUROC and AUPRC scores. While there was a slight drop in the model trained on 10,000 in terms of AUROC and AUPRC, it maintained the same balanced accuracy and a higher performance than SMOTE and original models. The performance gains using synthetic data for the random forest model were 0.082 in AUROC, 0.088 in AURPC, and 0.107 in Balanced Accuracy. The results are also visualized in Figure 2.

We also report the performance using SVMs and KNN models, where the models trained on the synthetic data outperformed SMOTE and the original models. For SVM models, we note that SMOTE achieved similar performance to the model trained on 1000 synthetic samples in terms of AUROC, but it was outperformed in terms of AUPRC and balanced accuracy, respectively. The models trained on synthetic data first outperformed the original model using 200 synthetic samples where the performance increased from 0.560 to 0.565 for the original model compared to the model trained using the synthetic data.

On the other hand, when using a KNN classifier, the performance of the models trained on SMOTE and the original data did not change across the three reported metrics, with an AUROC of 0.526, AUPRC of 0.255, and balanced accuracy of 0.500, respectively. We observe consistent performance gains for the model trained on 10,000 synthetic samples with a performance of 0.564 for AUROC, 0.272 for AURPC, and 0.569 for balanced accuracy respectively.

### 3.2. Interpretability Analysis

**Figure 3:**
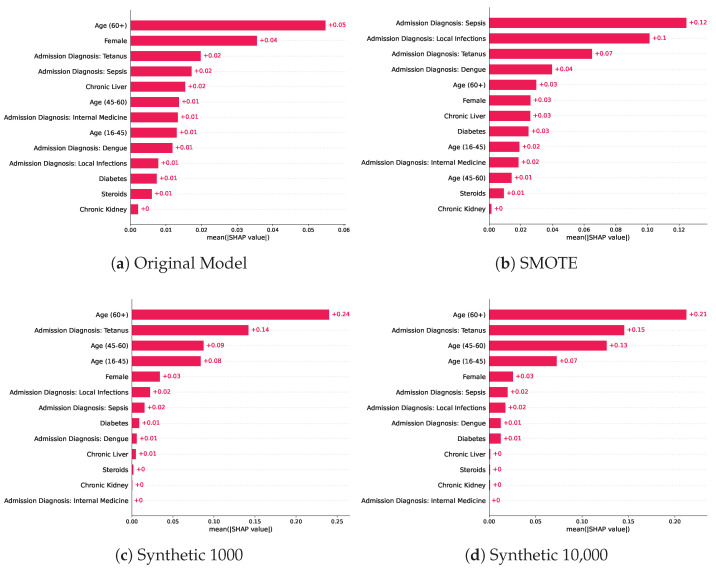
Mean absolute SHAP values across the baseline models trained on different training sets which included the original training set, SMOTE, and two models trained on synthetic datasets of various sizes.

The post-hoc SHAP interpretability analysis for the random forest models revealed the relative importance of the features in making predictions for each of the baseline models trained with various training sets, as shown in Figure 3. We chose the random forest models for this analysis as they achieved the highest score across the three evaluated classifiers, and we provide the SHAP analysis results of the two other classifiers in the Supplementary Material Figures S1 and S2, respectively. The most predictive features in the random forest model trained on the original data were a patient age > 60 years, female sex, and an admission diagnosis of Tetanus, which was in the top five for the models trained on synthetic datasets with the highest predictive feature being patient age > 60 years. The model trained using oversampled data using SMOTE, had a different order where patient age > 60 years ranked as the fifth most predictive feature after four features, indicating admission at diagnosis. The original model’s top five predictive features were patient age > 60 years, female sex, admission diagnosis of tetanus, and admission diagnosis of sepsis and chronic liver disease. On the other hand, the most predictive features for the model trained using oversampled training data via SMOTE were: admission diagnosis of Sepsis, admission diagnosis of local infections, admission diagnosis of Tetanus, admission diagnosis of Dengue, and patient age > 60 years. We notice that the synthetic model of 1000 patients had a different order of predictive features, where 4 out of 5 features were related to either sex or age and 1 indicated an admission diagnosis of tetanus. Similarly, the SHAP analysis of the highest performing model, trained on 10,000 synthetic samples, shows the patient age > 60 years as the most predictive feature followed by the admission diagnosis of tetanus, patient age 45–60 years, age 16–45 years, and female sex. The top predictive features for the models trained on synthetic samples were very similar with minor differences in the mean absolute SHAP value, which is also reflected in the similar performance in the predictive modelling tasks.

## 4. Discussion and Conclusions

Despite the increased research interest in using deep generative models, a gap exists in identifying the opportunities and limitations such models have in ML applications for low-source settings. To the best of our knowledge, this work is the first to investigate the use of deep generative models for generating EHRs from LMICs, where the datasets often come with small sizes and feature sets. Furthermore, our work validates the use of this synthetic data for real-world CDSS applications of high importance in LMICS, namely predicting HAI. Predicting HAI presents a challenge for clinicians since very limited data is collected from such settings. Improving prevention and treatment around HAI using CDSS is however a priority. Antibiotic resistance presents a global health challenge with an estimated death toll in 2019 alone, larger in magnitude than that of major diseases such as HIV and malaria [37]. LMICs tend to be one of the highest prescribers of antibiotics [38,39], yet they remain with limited antibiotic stewardship programs [12]. With the increased burden of HAI and its link to antimicrobial resistance, especially in LMICs, our work aims to fill a gap by developing simple CDSS to predict the probability of developing such infections despite the data scarcity. The proposed approach allows for improving diagnostic accuracy and performance without adding extra burden to the clinical staff involved with collecting more data, which is often not feasible. Furthermore, the interpretability component would allow for a better and more informed understanding of the risk scores predicted for each patient, towards machine learning transparency. The impact of a CDSS in providing early prediction to the clinical staff would allow the clinical staff to prioritize preventative strategies, optimise antimicrobial stewardship, as well as track care quality improvement, paving the way for better patient outcomes, with reduced operational costs related to hospital-acquired infections and patient deterioration.

While many papers have shown the feasibility of developing CDSS, very few discuss the challenges associated with deployment and real-world validation, especially in LMICs. Example challenges that are commonly discussed include integration in clinical workflows and medical staff adaption and decision-making process [40], security [41], and interoperability [42]. We believe that this work addresses an untapped area where data is scarce in terms of feature counts as well as the number of patients, which presents challenges for both the development and robust validation of CDSS. Another contribution of this work is demonstrating the impact of the size of the generated data on the performance of the predictive model, which we believe is an understudied area of research. We note that while many works investigated using deep generative models and synthetic datasets [43,44], to the best of our knowledge this is the first to investigate the impact of the synthetic dataset size with regards to EHRs applications.

In addition, several related works investigated the impact of using synthetic data in downstream tasks [44,45], but this work is the first to investigate the interpretability of models trained on synthetic data compared to other baselines such as SMOTE and the original training data. Older age and underlying medical conditions such as diagnosis at admission were identified as the most predictive features in both the original and models trained on synthetic datasets, which is also consistent with medical knowledge [46,47]. The interpretability analysis showed consistency in the ranking of the five most predictive features in the models trained on the synthetic samples, which is reflected in the similar predictive performance of the models trained on synthetic samples. Despite the comparable performance of the models trained on the original training samples and oversampled training data using SMOTE, we notice significant changes in the order and predictive value of features. For example, an admission diagnosis of local infections ranked as the ninth important feature for the original model, while it ranked as the second most predictive feature in the SMOTE model, compared to being the sixth and seventh most important feature for both models trained on synthetic baselines. In general, the order and SHAP values of the most predictive features of the models trained on the synthetic samples did not change when compared to the original model, where an admission diagnosis of Tetanus, female sex, and an age > 60 were the most contributors to the predictions, yet the model was able to achieve a higher performance, indicating its ability to preserve the predictive importance of features and data distribution.

This work also has several limitations. The results of the predictive models were not very high, which is related to the choice of using a simpler model to simulate a close setup to the target application setting in resource-constrained settings. Future works can investigate using more advanced models such as neural networks, and study the trade-off between computational complexity and impact on model performance. Another limitation of this work is related to the choice of the GAN model, where medGAN was used as one of the simpler and earlier works of GANs for EHRs. We believe that the results could be improved by using conditional variants of GANs [45] where the generation can be conditioned on a specific class or outcome, or other variants with more stable training such as Wasserstein GANs and boundary-seeking GANs [48,49] instead of the vanilla architecture where Jensen–Shannon Divergence (JSD) is used to learn the distribution of the data.

It is important to note that this work and the validation conducted are retrospective. Future works can investigate a prospective validation with a comparative analysis comparing the performance of models trained on the synthetic datasets concerning the model trained on the small original data. Such analysis would provide better insights to regulatory bodies on the approved models trained on syntactic data considering their impact on perspective deployment. Similarly, this work investigated the use of interpretability analysis as a way to study the underlying data distribution, however, future works could investigate the development of parsimonious models where a smaller set of features is used, which might result in significant reductions in the time associated with collecting the data.

The promising results of using synthetic data for training purposes will open the door for new research directions in building ML models for LMIC despite data scarcity, which can pave the way for new research and clinical decision support systems that best fit LMIC settings. Specifically, building tools that facilitate developing quick models with minimal data has great potential in increasing our understanding of rare and emerging diseases despite data scarcity, which in turn will help improve evidence-based practice [50] without increasing the burden on the clinical staff. Such efforts of synthetic data sharing will allow to bridge the gap in the CDSS for LMICs and evaluating the performance and feasibility, as well as fine-tuning the models built in developed countries in simulated settings without taking incurring deployment costs. Furthermore, in the absence of protection guidelines and regulations such as HIPAA [51] and GDPR [52] that are specific to low-resource settings, we believe that using deep generative models could encourage data owners in low-resource settings to share synthetic data for international research without compromising the privacy of patients coming from low-resource settings.

## Figures and Tables

**Table 1:** List of included patient features in terms of count and percentage prevalence in the population.

Co-Morbidities (n, %)	
Diabetes	35 (9.62%)
Steroids	15 (4.12%)
Chronic Liver	55 (15.11%)
Chronic Kidney	3 (0.82%)
**Demographics (n, %)**	
Female	242 (66.48%)
Age	
16–45	133 (36.54%)
45–60	142 (39.01%)
60+	89 (24.45%)
**Admission Diagnosis (n, %)**	
Tetanus	17 (4.67%)
Sepsis	45 (12.36%)
Local Infections	75 (20.60%)
Dengue	204 (56.04%)
Internal Medicine	139 (6.32%)
**Outcomes (n, %)**	
Hospital Acquired Infections	86 (23.6%)

**Table 2:** Results of the predictive model using the various baselines for training data. The results are reported in terms of AUROC, AUPRC, and balanced accuracy at a threshold of 0.5. Bolded numbers indicate the highest performance in each respective metric and estimator type.

Estimator	Model	AUROC	AURPC	Balanced Accuracy
Random Forest	Original	0.528 (0.386, 0.649)	0.246 (0.157, 0.377)	0.462 (0.389, 0.542)
	SMOTE	0.577 (0.428, 0.713)	0.281 (0.169, 0.451)	0.538 (0.419,0.651)
	Synthetic 200	0.511 (0.370,0.658)	0.261 (0.153, 0.431)	0.548 (0.448,0.648)
	Synthetic 500	0.533 (0.397,0.677)	0.266 (0.162, 0.440)	0.555 (0.459,0.657)
	Synthetic 1000	0.592 (0.455,0.723)	0.286 (0.185, 0.462)	0.548 (0.450, 0.661)
	Synthetic 2000	0.602 (0.459, 0.743)	0.295 (0.182, 0.469)	0.569 (0.471, 0.675)
	Synthetic 2500	**0.610 (0.460, 0.751)**	**0.334 (0.185, 0.542)**	0.569 (0.470, 0.669)
	Synthetic 10,000	0.605 (0.479, 0.742)	0.298 (0.191, 0.481)	**0.569 (0.477, 0.674)**
Support Vector Machines	Original	0.560 (0.418, 0.699)	0.267 (0.165, 0.434)	0.500 (0.500, 0.500)
	SMOTE	**0.568 (0.428, 0.707)**	0.270 (0.170, 0.419)	0.500 (0.500, 0.500)
	Synthetic 200	0.565 (0.427, 0.703)	0.285 (0.181, 0.454)	0.548 (0.452, 0.662)
	Synthetic 500	0.566 (0.427, 0.707)	0.287 (0.176, 0.459)	0.562 (0.470, 0.672)
	Synthetic 1000	**0.568 (0.436, 0.712)**	0.288 (0.185, 0.470)	0.548 (0.450, 0.659)
	Synthetic 2000	0.565 (0.431, 0.707)	0.286 (0.177, 0.457)	0.562 (0.470, 0.660)
	Synthetic 2500	0.564 (0.427, 0.690)	0.286 (0.178, 0.449)	0.562 (0.465, 0.671)
	Synthetic 10,000	0.565 (0.409, 0.708)	**0.292 (0.178, 0.460)**	**0.569 (0.476, 0.674)**
K-Nearest Neighbor	Original	0.526 (0.390, 0.666)	0.255 (0.154, 0.401)	0.500 (0.500, 0.500)
	SMOTE	0.526 (0.391, 0.657)	0.255 (0.157, 0.405)	0.500 (0.500, 0.500)
	Synthetic 200	0.528 (0.391, 0.675)	0.280 (0.167, 0.448)	0.548 (0.451, 0.650)
	Synthetic 500	0.520 (0.368, 0.669)	0.281 (0.168, 0.444)	0.555 (0.455, 0.662)
	Synthetic 1000	0.525 (0.386, 0.669)	0.281 (0.164, 0.445)	0.555 (0.465, 0.660)
	Synthetic 2000	0.542 (0.405, 0.687)	**0.290 (0.178, 0.457)**	0.555 (0.464, 0.669)
	Synthetic 2500	0.536 (0.394, 0.676)	0.281 (0.173, 0.437)	0.569 (0.469, 0.666)
	Synthetic 10,000	**0.546 (0.404, 0.689)**	0.272 (0.171, 0.441)	**0.569 (0.476, 0.675)**

## Data Availability

The data used in this work can be downloaded directly from The Oxford University Research Archive website (https://ora.ox.ac.uk/objects/uuid:fd5a236d-c739-48f1-8aae-09e2ae05a0a9) (accessed on 10 January 2023).

## Supplementary Materials

The following supporting information can be downloaded at: https://www.mdpi.com/article/10.3390/biomedicines11061749/s1, Figure S1: Mean absolute SHAP values across the baseline models trained on different training sets which included the original training set, SMOTE, and two models trained on synthetic datasets of various sizes; Figure S2: Mean absolute SHAP values across the baseline models trained on different training sets which included the original training set, SMOTE, and two models trained on synthetic datasets of various sizes; Table S1: The ranges considered for the hyperparameter search for the downstream predictive modelling section.

## Abbreviations

The following abbreviations are used in this manuscript:

EHRs	Electronic Health Records
ML	Machine Learning
LMICs	Low-Middle-Income Countries
SMOTE	Synthetic Minority Oversampling TEchnique
GANs	Generative Adversarial Networks
VAEs	Variational AutoEncoders
AUROC	Area Under the Receiver Operating Characteristic Curve
AUPRC	Area Under the Precision-Recall Curve

## References

[1] Kruk M.E., Gage A.D., Arsenault C., Jordan K., Leslie H.H., Roder-DeWan S., Adeyi O., Barker P., Daelmans B., Doubova S.V. (2018). High-quality health systems in the Sustainable Development Goals era: Time for a revolution. Lancet Glob. Health.

[2] Xiao C., Choi E., Sun J. (2018). Opportunities and challenges in developing deep learning models using electronic health records data: A systematic review. J. Am. Med. Inform. Assoc..

[3] Jeni L.A., Cohn J.F., De La Torre F. (2013). Facing imbalanced data–recommendations for the use of performance metrics. Proceedings of the 2013 Humaine Association Conference on Affective Computing and Intelligent Interaction.

[4] Van der Ploeg T., Austin P.C., Steyerberg E.W. (2014). Modern modelling techniques are data hungry: A simulation study for predicting dichotomous endpoints. BMC Med. Res. Methodol..

[5] Abbasgholizadeh Rahimi S., Cwintal M., Huang Y., Ghadiri P., Grad R., Poenaru D., Gore G., Zomahoun H.T.V., Légaré F., Pluye P. (2022). Application of artificial intelligence in shared decision making: Scoping review. JMIR Med. Inform..

[6] Dagliati A., Malovini A., Tibollo V., Bellazzi R. (2021). Health informatics and EHR to support clinical research in the COVID-19 pandemic: An overview. Briefings Bioinform..

[7] Adeloye D., Song P., Zhu Y., Campbell H., Sheikh A., Rudan I. (2022). Global, regional, and national prevalence of, and risk factors for, chronic obstructive pulmonary disease (COPD) in 2019: A systematic review and modelling analysis. Lancet Respir. Med..

[8] Baqui P., Marra V., Alaa A.M., Bica I., Ercole A., van der Schaar M. (2021). Comparing COVID-19 risk factors in Brazil using machine learning: The importance of socioeconomic, demographic and structural factors. Sci. Rep..

[9] Farran B., Channanath A.M., Behbehani K., Thanaraj T.A. (2013). Predictive models to assess risk of type 2 diabetes, hypertension and comorbidity: Machine-learning algorithms and validation using national health data from Kuwait—A cohort study. BMJ Open.

[10] Rudd K.E., Seymour C.W., Aluisio A.R., Augustin M.E., Bagenda D.S., Beane A., Byiringiro J.C., Chang C.C.H., Colas L.N., Day N.P. (2018). Association of the quick sequential (sepsis-related) organ failure assessment (qSOFA) score with excess hospital mortality in adults with suspected infection in low-and middle-income countries. JAMA.

[11] Mensah N.K., Boadu R.O., Adzakpah G., Lasim O.U., Amuakwa R.D., Taylor-Abdulai H.B., Chatio S.T. (2022). Electronic health records post-implementation challenges in selected hospitals: A qualitative study in the Central Region of southern Ghana. Health Inf. Manag. J..

[12] Galindo-Fraga A., Villanueva-Reza M., Ochoa-Hein E. (2018). Current challenges in antibiotic stewardship in low-and middle-income countries. Curr. Treat. Options Infect. Dis..

[13] Mills A. (2014). Health care systems in low-and middle-income countries. N. Engl. J. Med..

[14] Fernández A., Garcia S., Herrera F., Chawla N.V. (2018). SMOTE for learning from imbalanced data: Progress and challenges, marking the 15-year anniversary. J. Artif. Intell. Res..

[15] Kingma D.P., Welling M. (2013). Auto-encoding variational bayes. arXiv.

[16] Goodfellow I., Pouget-Abadie J., Mirza M., Xu B., Warde-Farley D., Ozair S., Courville A., Bengio Y. (2014). Generative adversarial nets. Adv. Neural Inf. Process. Syst..

[17] Ghosheh G., Li J., Zhu T. (2022). A review of Generative Adversarial Networks for Electronic Health Records: Applications, evaluation measures and data sources. arXiv.

[18] Thuy D.B., Campbell J., Nhat L.T.H., Hoang N.V.M., Hao N.V., Baker S., Geskus R.B., Thwaites G.E., Chau N.V.V., Thwaites C.L. (2018). Hospital-acquired colonization and infections in a Vietnamese intensive care unit. PLoS ONE.

[19] (2015). CDC and Prevention Surveillance Definitions for Specific Types of Infections. admin.inicc.org/media/2015-CDCNHSN-ALLDA-HAI-Definitions.pdf.

[20] Gholamy A., Kreinovich V., Kosheleva O. (2018). Why 70/30 or 80/20 Relation between Training and Testing Sets: A Pedagogical Explanation.

[21] Mi L., Shen M., Zhang J. (2018). A probe towards understanding gan and vae models. arXiv.

[22] Kwon Y.J., Toussie D., Azour L., Concepcion J., Eber C., Reina G.A., Tang P.T.P., Doshi A.H., Oermann E.K., Costa A.B. Appropriate Evaluation of Diagnostic Utility of Machine Learning Algorithm Generated Images. Proceedings of the PMLR 2020: Machine Learning for Health.

[23] Lee D., Yu H., Jiang X., Rogith D., Gudala M., Tejani M., Zhang Q., Xiong L. (2020). Generating sequential electronic health records using dual adversarial autoencoder. J. Am. Med. Inform. Assoc..

[24] Choi E., Schuetz A., Stewart W.F., Sun J. (2016). Medical concept representation learning from electronic health records and its application on heart failure prediction. arXiv.

[25] Qi Y. (2012). Random forest for bioinformatics. Ensemble Machine Learning: Methods and Applications.

[26] Noble W.S. (2006). What is a support vector machine?. Nat. Biotechnol..

[27] Larose D.T., Larose C.D. (2014). k-nearest neighbor algorithm. IEEE Trans. Syst. Man Cybern..

[28] Hajian-Tilaki K. (2013). Receiver operating characteristic (ROC) curve analysis for medical diagnostic test evaluation. Casp. J. Intern. Med..

[29] Ozenne B., Subtil F., Maucort-Boulch D. (2015). The precision–recall curve overcame the optimism of the receiver operating characteristic curve in rare diseases. J. Clin. Epidemiol..

[30] Mavrogiorgou A., Kiourtis A., Kleftakis S., Mavrogiorgos K., Zafeiropoulos N., Kyriazis D. (2022). A Catalogue of Machine Learning Algorithms for Healthcare Risk Predictions. Sensors.

[31] Zafeiropoulos N., Mavrogiorgou A., Kleftakis S., Mavrogiorgos K., Kiourtis A., Kyriazis D. (2023). Interpretable Stroke Risk Prediction Using Machine Learning Algorithms. Intelligent Sustainable Systems: Selected Papers of WorldS4 2022.

[32] Zou K.H., O’Malley A.J., Mauri L. (2007). Receiver-operating characteristic analysis for evaluating diagnostic tests and predictive models. Circulation.

[33] Ling C.X., Huang J., Zhang H. (2003). AUC: A better measure than accuracy in comparing learning algorithms. Proceedings of the Advances in Artificial Intelligence: 16th Conference of the Canadian Society for Computational Studies of Intelligence, AI 2003, Halifax, NS, Canada, 11–13 June 2003, Proceedings 16.

[34] Hancock J., Khoshgoftaar T.M., Johnson J.M. (2022). Informative evaluation metrics for highly imbalanced big data classification. Proceedings of the 2022 21st IEEE International Conference on Machine Learning and Applications (ICMLA).

[35] Lundberg S.M., Lee S.I. (2017). A unified approach to interpreting model predictions. Advances in Neural Information Processing Systems 30 (NIPS 2017).

[36] Lundberg S.M., Erion G.G., Lee S.I. (2018). Consistent individualized feature attribution for tree ensembles. arXiv.

[37] Murray C.J., Ikuta K.S., Sharara F., Swetschinski L., Aguilar G.R., Gray A., Han C., Bisignano C., Rao P., Wool E. (2022). Global burden of bacterial antimicrobial resistance in 2019: A systematic analysis. Lancet.

[38] Nguyen K.V., Thi Do N.T., Chandna A., Nguyen T.V., Pham C.V., Doan P.M., Nguyen A.Q., Thi Nguyen C.K., Larsson M., Escalante S. (2013). Antibiotic use and resistance in emerging economies: A situation analysis for Viet Nam. BMC Public Health.

[39] Nga D.T.T., Chuc N.T.K., Hoa N.P., Hoa N.Q., Nguyen N.T.T., Loan H.T., Toan T.K., Phuc H.D., Horby P., Van Yen N. (2014). Antibiotic sales in rural and urban pharmacies in northern Vietnam: An observational study. BMC Pharmacol. Toxicol..

[40] Improta G., Mazzella V., Vecchione D., Santini S., Triassi M. (2020). Fuzzy logic–based clinical decision support system for the evaluation of renal function in post-Transplant Patients. J. Eval. Clin. Pract..

[41] Lakshmanaprabu S., Mohanty S.N., Sheeba R.S., Krishnamoorthy S., Uthayakumar J., Shankar K. (2019). Online clinical decision support system using optimal deep neural networks. Appl. Soft Comput..

[42] Du Y., Rafferty A.R., McAuliffe F.M., Wei L., Mooney C. (2022). An explainable machine learning-based clinical decision support system for prediction of gestational diabetes mellitus. Sci. Rep..

[43] Choi E., Biswal S., Malin B., Duke J., Stewart W.F., Sun J. Generating multi-label discrete patient records using generative adversarial networks. Proceedings of the PMLR 2017: Machine Learning for Healthcare Conference.

[44] Esteban C., Hyland S.L., Rätsch G. (2017). Real-valued (medical) time series generation with recurrent conditional gans. arXiv.

[45] Li J., Cairns B.J., Li J., Zhu T. (2023). Generating synthetic mixed-type longitudinal electronic health records for artificial intelligent applications. NPJ Digit. Med..

[46] Kim B.G., Kang M., Lim J., Lee J., Kang D., Kim M., Kim J., Park H., Min K.H., Cho J. (2022). Comprehensive risk assessment for hospital-acquired pneumonia: Sociodemographic, clinical, and hospital environmental factors associated with the incidence of hospital-acquired pneumonia. BMC Pulm. Med..

[47] Chang Y.J., Yeh M.L., Li Y.C., Hsu C.Y., Lin C.C., Hsu M.S., Chiu W.T. (2011). Predicting hospital-acquired infections by scoring system with simple parameters. PLoS ONE.

[48] Baowaly M.K., Lin C.C., Liu C.L., Chen K.T. (2019). Synthesizing electronic health records using improved generative adversarial networks. J. Am. Med. Inform. Assoc..

[49] Engelmann J., Lessmann S. (2021). Conditional Wasserstein GAN-based oversampling of tabular data for imbalanced learning. Expert Syst. Appl..

[50] Palmer S., Jansen A., Leitmeyer K., Murdoch H., Forland F. (2013). Evidence-Based Medicine applied to the control of communicable disease incidents when evidence is scarce and the time is limited. Eurosurveillance.

[51] Centers for Disease Control and Prevention (2003). HIPAA privacy rule and public health. Guidance from CDC and the US Department of Health and Human Services. MMWR Morb. Mortal. Wkly. Rep..

[52] Voigt P., Von dem Bussche A. (2017). The EU General Data Protection Regulation (GDPR).

