# Supplementary material for: Synthesizing Electronic Health Records for Predictive Models in Low-Middle-Income Countries (LMICs)
Source: Biomedicines. 2023 Jun 18;11(6):1749. doi: 10.3390/biomedicines11061749 (PMC10295936; doi:10.3390/biomedicines11061749)
Supplement: Supplementary file 1 [file biomedicines-11-01749-s001.zip › biomedicines-2427240-supplementary.pdf]

Article

Synthesizing Electronic Health Records for predictive models in Low-Middle-Income Countries (LMICs)

Ghadeer O. Ghosheh[1]*, C. Louise Thwaites[2,3] and Tingting Zhu[1]

1 Department of Engineering Sciences, University of Oxford, United Kingdom
2 Oxford University Clinical Research Unit (OUCRU), Ho Chi Minh City, Vietnam
3 Centre for Global Health and Tropical Medicine, University of Oxford, United Kingdom
* Correspondence: ghadeer.ghosheh@eng.ox.ac.uk

Supplementary Material: Hyperparameter search

The searched hyperparameters for each of the models to predict HAIs are shown in Table S1. The final parameters were chosen using GridSearch.

Table S1. The ranges considered for the hyperparameter search for the downstream predictive modelling section.

Model	Hyperparameters	Values
**Random Forest**	N estimators	[100, 150,200]
**K-Nearest Neighbors**	N neighbors	[5,10,15,20]
	Power parameter	[1,2]
	Leaf size	[20,25,30,35,40,45,50]
**Support Vector Machine**	Kernel	[*poly*, *rbf*]
	Gamma	[1.e-02, 1.e+03]
	Regularization parameter C	[0.1, 1, 10, 100]

Supplementary Material: SHAP Analysis for SVM classifier

We conducted an interpretability analysis for the SVM classifier. The SHAP values for the baselines and selected models trained on synthetic data are shown in Figure S1.

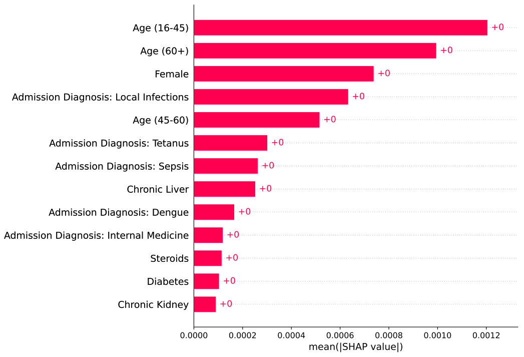

(a) Original Model

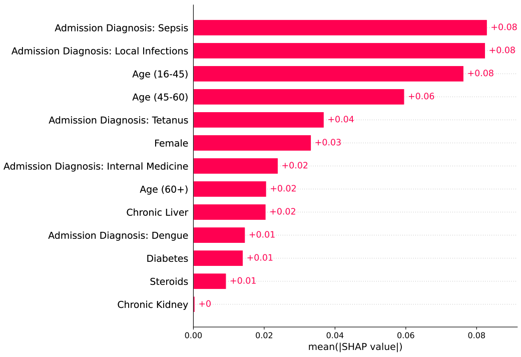

(b) SMOTE

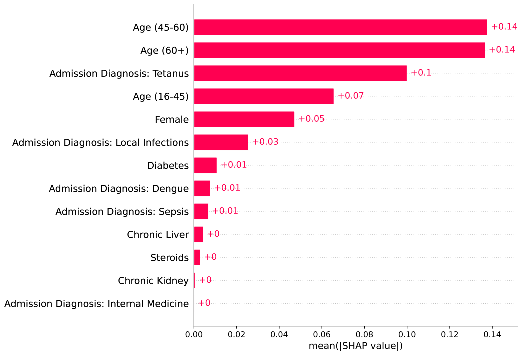

(c) Synthetic 1000

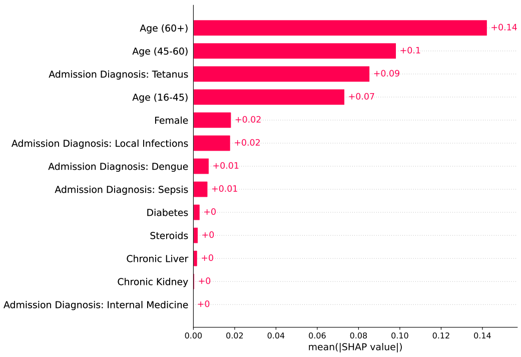

(d) Synthetic 10000

Figure S1. Mean absolute SHAP values across the baseline models trained on different training sets which included the original training set, SMOTE, and two models trained on synthetic datasets of various sizes.

Supplementary Material: SHAP Analysis for KNN classifier

We conducted an interpretability analysis for the KNN classifier. The SHAP values for the baselines and selected models trained on synthetic data are shown in Figure S2.

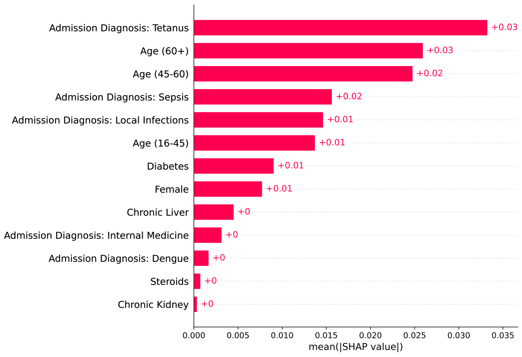

(a) Original Model

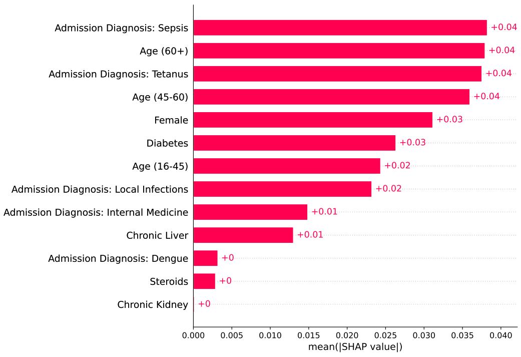

(b) SMOTE

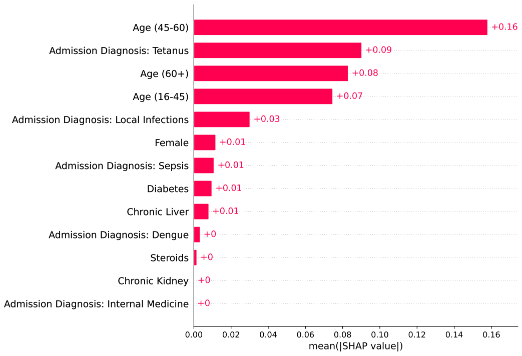

(c) Synthetic 1000

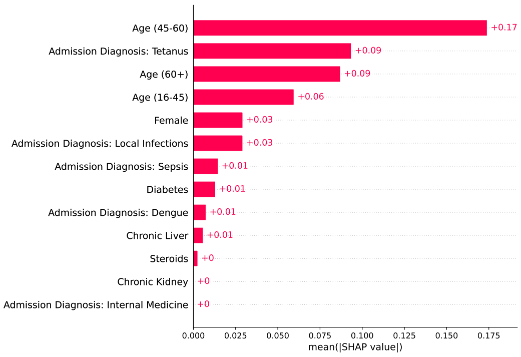

(d) Synthetic 10000

Figure S2. Mean absolute SHAP values across the baseline models trained on different training sets which included the original training set, SMOTE, and two models trained on synthetic datasets of various sizes.
